# Percutaneous bone-anchored hearing implant surgery: inside or outside the line of incision?

**DOI:** 10.1007/s00405-016-4020-7

**Published:** 2016-04-16

**Authors:** Ruben M. Strijbos, Christine A. den Besten, Emmanuel A. M. Mylanus, Myrthe K. S. Hol

**Affiliations:** Department of Otorhinolaryngology, Radboud University Medical Center, Post 377, PO box 9101, 6500 HB Nijmegen, The Netherlands

**Keywords:** BAHA, Bone-anchored hearing implant, Surgical technique, Linear incision, Soft tissue reactions, Hearing loss

## Abstract

The objective of this historical cohort study was to compare soft tissue reactions in adults after bone-anchored hearing implant (BAHI) surgery when the percutaneous implant is placed inside or outside the line of incision. All adult patients who received a percutaneous BAHI between 1 January 2010 and 31 January 2014 in our tertiary referral centre were identified. Patients were selected if operated by two surgeons, who perform the same standardised linear incision technique with one of them placing the implant outside the incision while the other prefers placement inside the line of incision. A total of 202 patients and 211 implants were included in the case analysis. The results showed the registration of a soft tissue reaction Holgers ≥1 in 47 implants (49.0 %) placed outside the incision compared to 70 implants (60.9 %) which were placed inside the line of incision. An adverse soft tissue reaction, Holgers ≥2, was noticed in 17 implants (17.7 %), respectively, 20 implants (17.4 %). No significant differences were found between the two groups for both the presence of soft tissue reactions Holgers ≥1 (*p* = 0.322) and a Holgers score ≥2 (*p* = 0.951). During the follow-up three implants were lost (1.4 %) and in 18 of 211 implants one or multiple revisions were performed (8.5 %). In conclusion, this study did not show any differences in the presence of postsurgical (adverse) soft tissue reactions between placement of the percutaneous BAHI inside or outside the line of incision.

## Introduction

Since Tjellström introduced the percutaneous bone-anchored hearing implant (BAHI) for bone conduction hearing in 1977; two hundred thousand patients have already benefited from this hearing rehabilitation option. A bone conduction device (BCD) is a successful treatment for patients with both conductive and mixed hearing loss [1, 2] and single-sided deafness [3–6]. The procedure for implantation of osseointegrated implants is safe with a lack of major complications [7, 8]. Nevertheless, adverse soft tissue reactions around the titanium skin-penetrating implant are still a frequent problem, leading to discomfort for the patient and increased visits to the outpatient clinic. A small percentage of these patients will suffer from recurrent soft tissue problems, soft tissue overgrowth or even implant loss [7–11]. The classification proposed by Holgers et al. in 1988 is the most commonly used grading system for these postsurgical skin reactions [9].

**Fig. 1:**
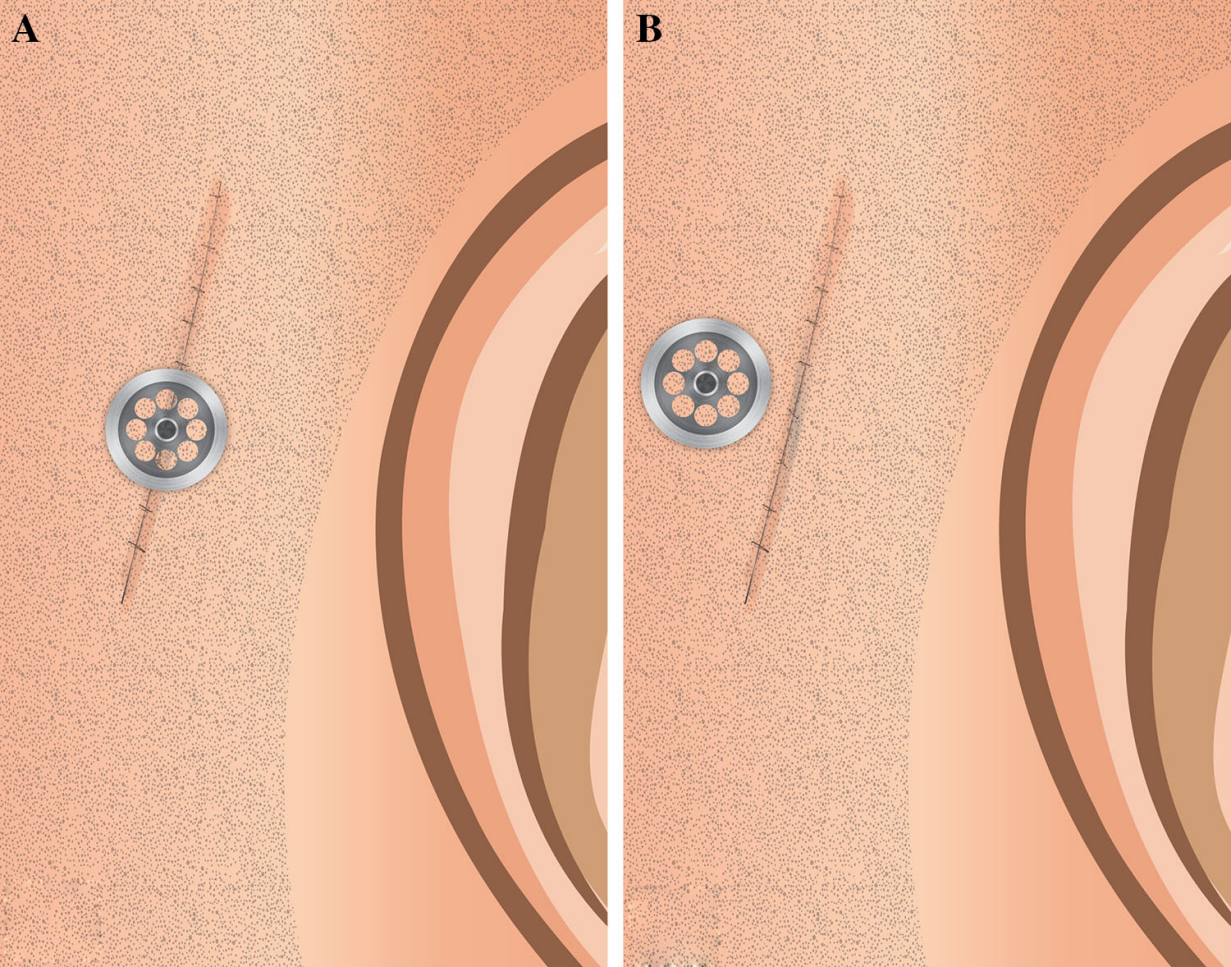
**a** Linear incision technique with placement of the percutaneous abutment outside the line of incision. **b** Linear incision technique with placement of the percutaneous abutment inside the line of incision

Over the years there have been various surgical techniques used for bone-anchored hearing implantation to prevent and minimise skin problems postoperatively, like the free retro-auricular full-thickness skin graft, pedicled grafts, dermatome technique and the linear incision technique [12, 13]. The linear incision technique has become most popular because of its procedural simplicity and association with less skin complications compared to the other techniques [13, 14]. This technique has become even more popular nowadays with so-called soft tissue preservation, in which after the linear incision no reduction of subcutaneous tissue is performed. The remaining item to address is the implant placement when using the linear incision technique, i.e. the implant inside the line of incision or the implant outside the line of incision (Fig. 1). It is suggested that when placing the implant outside of the incision, it would be surrounded by scarcely traumatised skin, reducing the inflammatory reaction occurring around it and leading to less skin complications [15].

The aim of the current study is to identify if there is a difference in postsurgical soft tissue reactions, as classified by the Holgers grading system, in adults when the percutaneous titanium implant is placed inside or outside the line of incision.

## Methods

### Patients

For this cohort study, all adult patients (aged 18 years or older) who received any type of percutaneous BAHI at our clinic between 1 January 2010 and 31 January 2014 were identified from our Bone Implant database. Patients operated by two surgeons, A and B, were selected. Both surgeons use the same standardised linear incision technique; they were trained and work in the same centre. Surgeon A places the implant outside the line of incision on a consistent basis, while the other surgeon B consistently uses the technique with placement of the implant inside the line of incision.

Eligibility criteria were: one staged procedure with tissue reduction, initial placement of the implant (no previous implant loss or removal) and availability of the medical record including at least one postoperative visit at our outpatient clinic.

### Surgical techniques and post-surgery protocol

In the selected study period the simplified linear incision technique with subcutaneous soft tissue reduction was consistently used [14]. In this procedure, a longitudinal incision of approximately 30 mm is made with the optimal site of implantation being approximately 50 to 55 mm posterosuperiorly to the ear canal. The next step is the exposure and mobilisation of the periosteum after sharp dissection of the subcutaneous tissue. Subsequently, the implant is placed and there will be resection of subcutaneous tissue over an area of approximately 2 cm around the incision. The remaining periosteum will be removed. In the final step of the surgical procedure, surgeon A punches the skin next to the incision while surgeon B punches the skin in the line of incision, consequently placing the implant outside or inside the line of incision, respectively .

The first postoperative visit was a week after surgery, when the healing cap and gauze with antibiotic ointment were removed, followed by an inspection of the incision. All patients received topical therapy with hydrocortison/oxytetracycline/polymyxine B for 2 weeks during the first postoperative visit. This visit was followed by an appointment for fitting of the sound processor after 3–6 weeks. Further follow-up was in general after 3 and after 12 months. Extra visits could be initiated by physicians or patients depending on arising problems or individual needs. In addition, some patients visited the outpatient clinic more often because they participated in clinical trials [16, 17]. At each visit, there was registration of the degree of skin reaction, using the Holgers grading system, and therapeutic intervention if applicable [9].

### Case analysis

Data were obtained from our Bone Implant database and patients medical charts. Information about incision technique, surgeon and implant type was collected. Unless otherwise described in the operative report, it was registered that surgeon A placed the implant outside the incision line and surgeon B placed the implant inside the line of incision.

All follow-up visits by one of our physicians, residents or specialised nurses were included in the analysis; consultation by telephone was not included. The notes from the physical examination were used to determine the presence and timing of a soft tissue reaction. A Holgers classification 2 or higher was registered as an adverse soft tissue reaction and a Holgers classification of 1 or higher was classified as a soft tissue reaction. The reason for this distinction was because of the clinical consequences of the adverse soft tissue reaction, namely an indication for (local) treatment. Additionally, if the Holgers notation was missing but there was notation of any redness, swelling, moistness and/or granulation around the titanium skin-penetrating implant in the medical record, this was still interpreted as presence of a soft tissue reaction. A soft tissue reaction was considered not present in case of a Holgers score 0 or no notation of inflammation of the skin in the notes of the physical examination. A lack of description about the tissue surrounding the implant in the notes of a follow-up contact was considered as missing data.

Therapeutic interventions for skin problems were recorded per visit. Conforming to the general protocol in our hospital, all patients received topical therapy with hydrocortison/oxytetracycline/polymyxine B during the first weeks post-surgery. This topical therapy was therefore not considered as a therapeutic intervention in our study. Alternative therapeutic interventions were distinguished: topical antibiotic ointment, healing cap replacement and revision surgery (change of the abutment and/or soft tissue revision). Implant loss was registered as well.

Finally, the background characteristics mental retardation, dermatological disease and diabetes mellitus were registered, because recent studies focus on identification of these comorbidities as possible risk factors in the context of soft tissue reactions after BAHI surgery [7, 18–20]. If there were no notes for these conditions in the medical chart or any correspondence within the chart of the patient, this comorbidity factor was considered absent. End of the follow-up was defined as the last visit before March 2015.

### Statistical analysis

The presence of postsurgical soft tissue reaction during follow-up in the two groups was analysed using Kaplan–Meier curves. A log-rank test was performed to determine differences in soft tissue reaction between the cohorts. The level of significance applied was *p* = 0.05. All analyses were performed using Statistical Package for Social Sciences (IBM SPSS Statistics for Windows, Armonk, NY; IBM Corp), version 20.0.

## Results

### Patients

A total of 202 patients and 211 implants could be included in the cohort in the period from 1 January 2010 until 31 January 2014. Surgeon A placed the implants consistently outside the line of incision and surgeon B placed the implants consistently inside the line of incision. Three exceptions were retrieved, in which the purpose always was to place the implant inside the line of incision. However, after closure, due to anatomical variation, it turned out the implant was outside the line of incision. From all implants, 96 BAHIs were placed outside the line of incision. The mean age in this group was 55 years (range 18–85 years, SD ± 16) and the median follow-up was 653 days per implant [interquartile range (IQR) 337–1058 days]. There were 115 implants placed inside the line of incision. The mean age was 53 years (range 18–83 years, SD ± 15) and the median follow-up was 548 days per implant (IQR 353–1046 days). A number of 81 of 202 patients participated in a clinical trial with a more extensive (standard) follow-up protocol, similarly distributed over the two different cohorts. All the baseline characteristics of the patient population are shown in Table 1. No significant differences in these baseline characteristics between both groups were noticed. The use of longer abutments was equally distributed between the study groups, however, slightly more previous generation implants and abutments were used in the inside group. In Table 2 the other surgical characteristics are summarised. The length of all implants was 4 mm.

**Table 1:** Background characteristics of the patient population

	Inside		Outside	
	*n*	%	*n*	%
Total patients	111	100	92	100
Total implants	115		96	
Gender				
Male	43	38.7	38	41.3
Female	68	61.3	54	58.7
Age at surgery				
Mean (years) [±SD]	53 [±15]		55 [±16]	
Range (years)	18–83		18–85	
Aetiology of hearing loss				
Acquired conductive/mixed hearing loss	74	66.7	73	79.3
Congenital conductive hearing loss	9	8.1	5	5.4
Single-sided deafness	28	25.2	14	15.2
Comorbidity factors				
Mental retardation	5	4.5	3	3.3
Diabetes mellitus	10	8.9	7	7.6
Dermatological disease	10	8.9	9	9.8

**Table 2:** Surgical characteristics of the patient population

	Inside		Outside	
	*n*	%	*n*	%
Total implants	115	100	96	100
Follow-up				
Median (days)	548		653	
Interquartile range (days)	353–1046		337–1058	
Loading time				
Mean (weeks) [±SD]	5.5 [±3.2]		5.4 [±3.0]	
Type of implant-abutment				
Previous generation Cochlear	14	12.2	5	5.2
BIA210	9	7.8	3	3.1
BIA300	36	31.3	35	36.5
BIA400	0	0	1	1.0
Ponto Regular	32	27.8	37	38.5
Ponto Wide	24	20.9	15	15.6
Abutment length				
5.5 mm	22	19.1	7	7.3
6 mm	74	64.3	72	75.0
8.5 mm	1	0.9	0	0
9 mm	14	12.2	10	10.4
10 mm	0	0	1	1.0
Unknown	4	3.5	6	6.3

### Implant loss and revision surgery

Three implants were lost during complete follow-up (1.4 %). All these implants were placed outside the line of incision. One implant was lost 3 days after surgery. The medical chart reported a poor quality of the temporal bone. The other implants were lost after 46 days and after more than 3 years (this patient suffered from recurrent infections with peri-implantitis in the period prior to implant loss).

During the complete follow-up, in 18 of 211 implants, one or multiple revisions were performed (8.5 %). In the group with the implant outside the line of incision, revision surgery was performed in 5 of 96 implants (5.2 %). In the set of implants placed inside the line of incision, revision surgery was undertaken for 13 of 115 implants (11.3 %). This difference in performed revision surgery between both groups, as calculated with a log-rank test, was not significant (*p* = 0.129). An overview of the revision surgery and other therapeutic interventions in both groups is given in Table 3.

**Table 3:** Overview of therapeutic interventions and revision surgery during follow-up

	Inside		Outside	
	*n*	%	*n*	%
Number of local treatments				
0	62	53.9	55	57.3
1	39	33.9	30	31.3
2	9	7.8	7	7.3
3	4	3.5	2	2.1
4	1	0.9	1	1.0
5	0	0	0	0
6	0	0	1	1.0
Number of systemic treatments				
0	112	97.4	94	97.9
1	3	2.6	1	1.0
2	0	0	1	1.0
Revision surgery^a^				
Soft tissue reduction	4		3	
Secondary higher abutment	7		2	
New implant	1		0	
Both soft tissue reduction + higher abutment	3		0	
Both higher abutment + new implant	1^b^		0	

^a^Regarding the group with implants placed inside the line of incision: in three implants was two times revision surgery performed, numbers shown indicate how often the procedure is performed

^b^During this revision procedure was the implant accidently lost while removing the previous abutment, so both a higher abutment and a new implant were placed

### Soft tissue reaction

The outcome was divided in the presence of a soft tissue reaction (i.e. Holgers grade 1 or higher) and the presence of an adverse soft tissue reaction (i.e. Holgers grade 2 or higher). In 6.7 % of the follow-up contacts a notation of a soft tissue reaction was available but no Holgers classification was given, and in 3.7 % of the follow-up contacts a description about the tissue surrounding the implant was missing. A soft tissue reaction Holgers ≥1 was noticed in 47 implants (49.0 %) when the implant was placed outside the line of incision compared to 70 implants (60.9 %) which were placed inside the line of incision. The median time until the first soft tissue reaction was 90 days (IQR 21–366 days) and 95 days (IQR 44–344 days), respectively.

An adverse soft tissue reaction, Holgers grade ≥2, was registered in 17 implants (17.7 %) when the implant was placed outside the line of incision. In the group of implants placed inside the line of incision, 20 implants (17.4 %) presented with a Holgers ≥2. The median time until the first adverse soft tissue reaction was 363 days (IQR 127–675 days) and 183 days (IQR 112–370 days), respectively.

**Fig. 2:**
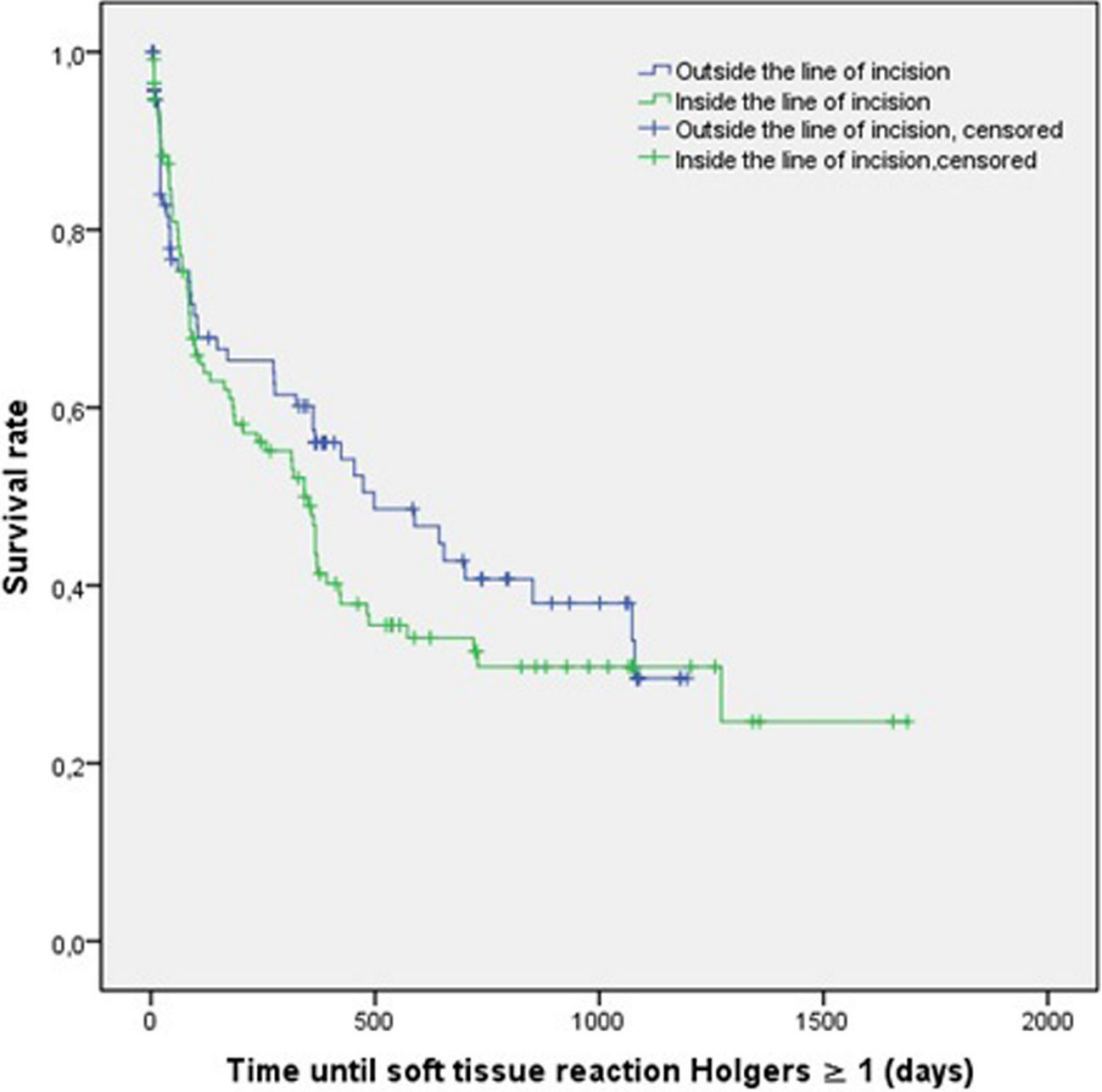
Kaplan–Meier analysis: soft tissue reaction Holgers ≥1

**Fig. 3:**
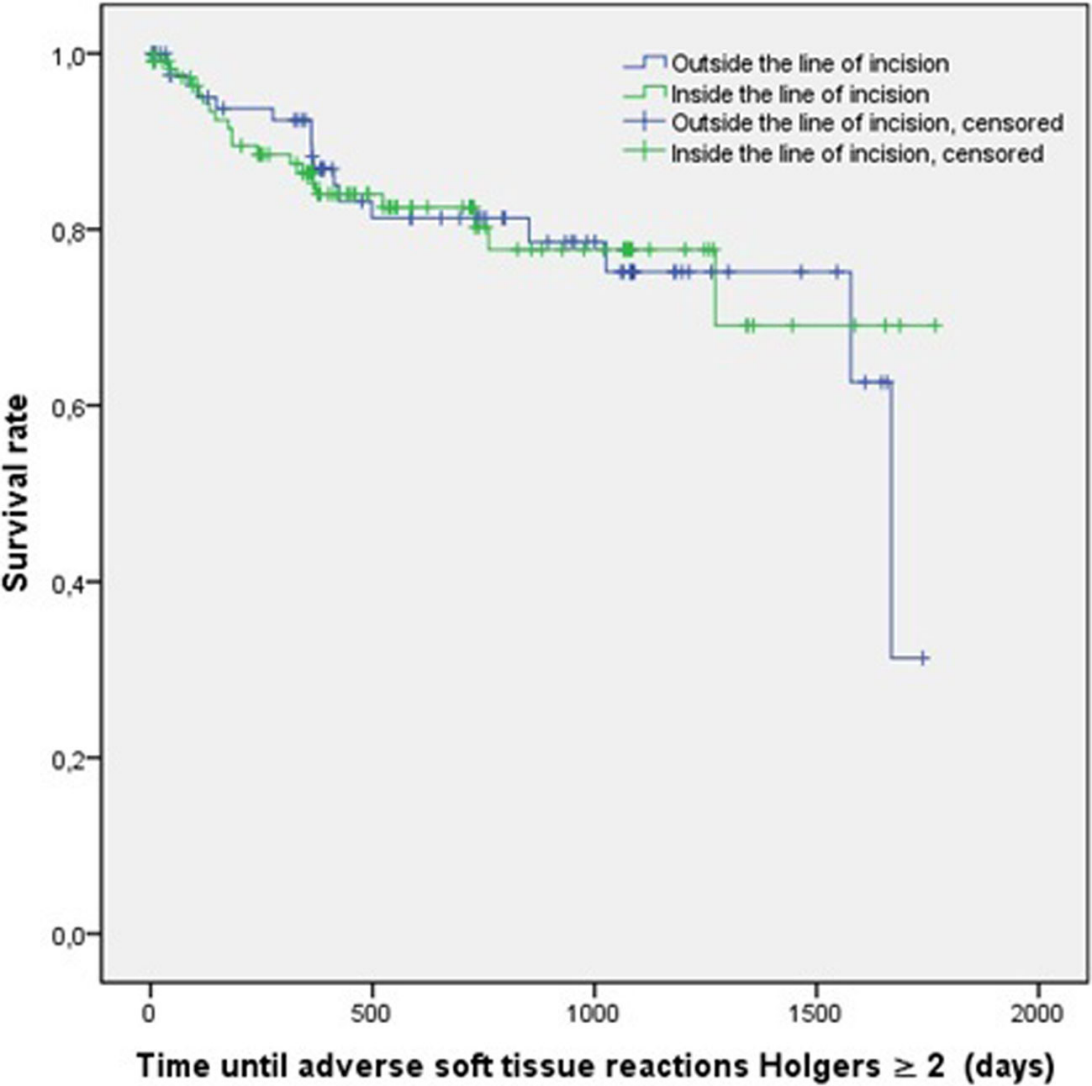
Kaplan–Meier analysis: adverse soft tissue reaction Holgers ≥2

For both outcome measures a survival curve was calculated by the Kaplan–Meier method; the Kaplan–Meier curves and survival tables are shown in Figs. 2 and 3 and Tables 4 and 5. The Kaplan–Meier curves show the probability of surviving, i.e. not encountering an (adverse) soft tissue reaction, in a given length of time. The corresponding survival tables provide additional information about the cumulative events (CE), remaining cases (RC) and cumulative proportion surviving (CPS) at given points in the time during the follow-up. In these tables, the cumulative events are defined as the number of implants with (adverse) soft tissue reactions and the remaining cases are the implants still in the follow-up without soft tissue problems. The term cumulative proportion surviving can be explained as a statistical representation of the proportion of implants that have not reached the terminal event (i.e. skin reaction) by the end of an interval. A log-rank test was executed to compare the survival curves of the two surgical techniques. No significant differences were found between the two groups for both the presence of soft tissue reactions (*p* = 0.322) and a Holgers score of 2 or higher (*p* = 0.951) during the follow-up.

**Table 4:** Survival table soft tissue reactions Holgers ≥1

	3 months			6 months			12 months			24 months			36 months		
	CE	RC	CPS	CE	RC	CPS	CE	RC	CPS	CE	RC	CPS	CE	RC	CPS
Inside	35	72	0.678 (0.045)	42	63	0.610 (0.047)	56	43	0.468 (0.049)	69	18	0.309 (0.049)	69	7	0.309 (0.049)
Outside	24	57	0.716 (0.049)	29	51	0.653 (0.052)	35	42	0.574 (0.055)	44	20	0.407 (0.061)	47	3	0.296 (0.072)

*CE* cumulative events, *RC* remaining cases, *CPS* cumulative proportion surviving (SE)

**Table 5:** Survival table soft tissue reactions Holgers ≥2

	3 months			6 months			12 months			24 months			36 months		
	CE	RC	CPS	CE	RC	CPS	CE	RC	CPS	CE	RC	CPS	CE	RC	CPS
Inside	3	102	0.973 (0.016)	9	94	0.914 (0.027)	14	72	0.864 (0.034)	18	35	0.802 (0.044)	19	14	0.777 (0.050)
Outside	3	76	0.963 (0.021)	5	72	0.937 (0.027)	9	63	0.883 (0.037)	13	39	0.813 (0.048)	15	14	0.752 (0.061)

*CE* cumulative events, *RC* remaining cases, *CPS* cumulative proportion survival (SE)

## Discussion

In this historical cohort study, 202 patients and 211 implants were studied with a total median follow-up time of 555 days (IQR 351–1055). No significant differences were found in the presence of postsurgical soft tissue reactions or adverse soft tissue reactions between the two cohorts, i.e. the placement of the percutaneous BAHI inside or outside the line of incision.

As stated in the “Introduction”, of all possible techniques for placement of bone-anchored hearing implants the linear incision technique is most popular because of its favourable outcomes [13, 14]. Nevertheless, little is known about the placement of the BAHI inside or outside the line of incision, as both techniques are described and used. To our knowledge, this is the first large-scale retrospective study focusing on this particular step in the procedure of implantation with the linear incision technique. Although this retrospective study design and a setting in a tertiary referral centre made it possible to include a relatively large cohort of patients, it might be possible both groups lack patients to detect somewhat smaller differences in the presence of skin reactions.

In addition to the large cohort investigated in this study, other strengths are the representative characteristics of our sample. The rates of implant loss and revision surgery were similar or slightly better compared with previous studies in our centre [7, 20] or according to other studies [8, 13]. In addition, no differences in baseline characteristics between both groups were noticed.

Despite the fact that the follow-up contacts in the medical charts had few missing data (3.7 %), the retrospective study design could be considered as a limitation of this study. All data were obtained from our Bone Implant database and patients medical charts and during this case analysis, as described in the “Methods”, assumptions were made. First of all, in 6.7 % of the follow-up contacts, only a description of the skin surrounding the titanium skin-penetrating implant was documented without a Holgers classification. In these cases, any notation of signs of inflammation was registered as the presence of a soft tissue reaction (i.e. Holgers grade 1 or higher). If there was no notation of inflammation of the skin, it was interpreted as the absence of a soft tissue reaction (i.e. Holgers grade 0). Moreover, it was chosen to exclude consultation by telephone, because these soft tissue reactions could not be objectified and graded by trained professionals. However, this decision could cause an underestimation of the amount of postoperative skin problems. Furthermore, for some of the background characteristics frequently incomplete patient information was available in the charts.

Another limitation could be confounding caused by the two surgeons performing in principle only one of the surgical techniques for implantation (surgeon A: implant outside the line of incision, surgeon B: implant inside the line of incision). Although the other steps in the surgical procedure were similar, it is inevitable some minor differences in the surgical and peri-operative approaches are present, possibly influencing the outcomes. Ideally, both surgeons should have been performing both the surgical techniques to prevent this confounding factor. This is a limitation of the study design. Moreover, slightly more previous generation implants and abutments were present in the group with implants placed inside the line of incision. This also might have been a confounding factor, because ongoing developments in the field of implants and abutments have led to less skin reactions in the current types [21].

Finally, the duration of the follow-up of the implants was limited with a median of 653 days (IQR 337–1058 days) and 548 days (IQR 353–1046 days) for implants placed outside and inside the line of incision, respectively . Nevertheless, based on our hypothesis it was expected that differences between both techniques would be seen shortly postoperative, so this relative restricted difference in follow-up was not considered as a serious limitation. In the context of follow-up contacts, it was noticed that 48 patients, slightly unequally divided between the two groups, had less than three follow-up contacts. This can only partially be explained by the group of patients which had received the BAHI most recently. Other reasons might be that patients did not encounter any problems postoperatively, completed their follow-up at another clinic or did not use the BAHA because of (skin) problems. This could influence the outcome positively or negatively.

Future research should be focusing on the sustainability of these already clinically favourable results with new generation implants and abutments. This is also relevant in the context of modifications in the linear incision, for example the linear incision technique with tissue preservation. It has been advocated that this less invasive approach results in faster healing, better aesthetic appearance and less soft tissue problems. Due to the development of longer abutments, it has been possible to study this proposed modification in the clinical practise. Several recent prospective studies have already shown promising outcomes compared to the traditional technique [22–26]. It has been suggested in the tissue preservation technique to preferably place the implant outside the line of incision. In the light of the outcomes of this evaluation, also in tissue preservation the implant position might not be influencing the outcomes. Additionally, since these implants are also an important hearing rehabilitation option in children, it would be interesting to find out if our results are also valid for this population, especially because implantation in children is more vulnerable to skin problems postoperatively compared to adults [7, 8, 11].

In conclusion, no significant difference was found in the presence of soft tissue reactions and adverse soft tissue reaction (i.e. Holgers grade 2 or higher) between the placement of the BAHI inside or outside the line of incision. In the procedure of the linear incision technique used in titanium percutaneous osseointegrated hearing implants for bone conduction hearing, both placing the implant inside and outside the line of incision can be used depending on the surgeons’ experience and preferences. In the area of ongoing developments in the surgical procedure, with the goal to further minimise skin problems postoperatively, this study contributes to the knowledge that is available to date.
